# Rescuing Ischemic Brain Injury by Rewiring Mitochondrial Electron Flow

**DOI:** 10.1101/2025.05.19.650489

**Published:** 2025-05-23

**Authors:** Belem Yoval-Sánchez, Ivan Guerrero, Qiuying Chen, Sergei Sosunov, Fariha Ansari, Max Siragusa, Csaba Konrad, Zoya Niatsetskaya, Anna Stepanova, Anatoly A. Starkov, Sergei Khruschev, Jordi Magrane, Arina A. Nikitina, Oxana Bereshchenko, Ping Zhou, LiPing Zhou, Marten Szibor, Ilka Wittig, Giovanni Manfredi, Steven S. Gross, Vadim Ten, Alexander Galkin

## Abstract

Mitochondrial metabolic flux alterations are critical drivers of acute ischemia-reperfusion (IR) brain injury. Reverse electron transfer (RET), defined as the upstream flow of electrons from the quinone pool to complex I, is a major source of pathological reactive oxygen species (ROS) under stress conditions. Using an *in vivo* model of brain IR, we show that RET-supporting substrates – succinate and glycerol 3-phosphate – accumulate during oxygen deprivation. Rapid oxidation of these substrates by brain mitochondria upon reoxygenation drives massive ROS production, while also leading to over-reduction and dissociation of the complex I flavin mononucleotide (FMN) cofactor. The resulting FMN-deficient complex I becomes catalytically impaired, unable to oxidize NADH or to produce ROS.

To mitigate RET and preserve complex I function, we used transgenic mice xenotopically expressing alternative oxidase (AOX). This enzyme bypasses complexes III and IV by directly oxidizing the reduced quinone pool and passing electrons onto molecular oxygen. AOX expression did not alter complex I abundance, supercomplexes assembly, or basal respiration rates, but effectively diverted electrons from the quinone pool, decreasing RET flux via complex I and limiting ROS generation during IR. This attenuation of RET preserved complex I FMN binding, suppressed oxidative stress, and conferred neuroprotection *in vivo* . Our findings reveal a novel strategy for rewiring mitochondrial electron flux to mitigate initial IR brain injury, highlighting modulation of the quinone pool by AOX as a potential therapeutic strategy for IR.

## Full Text Availability

The license terms selected by the author(s) for this preprint version do not permit archiving in PMC. The full text is available from the preprint server.

